# Carcinogenesis: An Alternative Hypothesis Comparing Mutagenic Versus Metabolic Models

**DOI:** 10.3390/biology14101314

**Published:** 2025-09-23

**Authors:** Albert Alhatem, Claude E. Gagna, Muriel W. Lambert, Emily Keenan, W. Clark Lambert

**Affiliations:** 1 Departments of Pathology and Dermatology, Rutgers-New Jersey Medical School, Newark, NJ 07103, USA; cgagna@nyit.edu (C.E.G.); mlambert@njms.rutgers.edu (M.W.L.); elk75@njms.rutgers.edu (E.K.); 2 Department of Biological and Chemical Sciences, New York Institute of Technology, Old Westbury, NY 11568, USA

**Keywords:** carcinogenesis, mutations, metabolic reprogramming, Warburg effect, oxidative phosphorylation, atavistic response, cell migration, angiogenesis

## Abstract

Carcinogenesis, while traditionally attributed to the accumulation of driver mutations in genes regulating cell proliferation and apoptosis, may also be explored as a consequence of fundamental metabolic reprogramming, an idea catalyzed by the Warburg effect, where cancer cells exhibit a paradoxical preference for glycolysis over the far more efficient oxidative phosphorylation. This implies that metabolic dysregulation may be a primary instigator of neoplastic transformation. Our hypothesis proposes that the abrupt loss of cellular energy may stimulate an atavistic response, wherein rapid proliferation and migration are triggered to enhance survival in fluctuating environments. These responses lead to pathological angiogenesis and unchecked cell growth, thereby bridging the gap between genetic and metabolic pathways of carcinogenesis.

## Simple Summary

For decades, scientists believed that cancer started mainly because of gene mutations. However, what if cancer doesn’t always begin with bad genes? We explore another possibility: that cancer might actually begin when cells lose their ability to make energy properly. When this happens, cells may “panic” and switch on an ancient survival mode from the time when we were single-celled organisms. In this mode, cells start multiplying and moving, even if it is harmful in today’s human body. This reaction, meant to help in emergencies like low oxygen or energy, can lead to the chaotic growth seen in cancer. We suggest that this energy crisis might come before the gene mutations we usually blame, making metabolic failure a key trigger of cancer. If this idea is right, new ways to treat or even prevent cancer could be found by focusing not just on genes, but on how cells use energy.

## 1. Two Competing Models for Carcinogenesis

Genomic analyses of diverse cancers have identified recurrent “driver mutations” in genes regulating cell proliferation, seemingly supporting the view that such mutations constitute initiating events in oncogenesis [1,2,3,4,5]. This interpretation assumes a linear progression from genetic alteration to malignant transformation. However, Otto Warburg, the noted German biochemist and Nobel Laureate, through biochemical investigations observed that many cancers preferentially utilize the pentose phosphate pathway over the tricarboxylic acid (TCA) cycle for energy production [6,7,8,9,10,11,12,13,14,15,16], a metabolic shift now exploited in positron emission tomography (PET) imaging [17], in which metastatic cancer is identified radiographically [17].

Warburg hypothesized that carcinogenesis originates primarily from metabolic dysfunction rather than mutations in proliferative pathways [8]. This long-standing dichotomy persists. Yet, we propose a unifying framework that reconciles these perspectives, integrating contemporary findings to inform novel directions in cancer research and therapy, while acknowledging the multifaceted nature of tumorigenesis [4]. We do not suggest that all cancers arise in this manner, just that a significant number do [4,9]. Carcinogenesis is a complex, many faceted processes [4]; we do not suggest otherwise.

We believe that some genetic alterations are inferred to be initiating events in oncogenesis, leading to the accumulation of subsequent mutations [1,2,3,4,5]. Their identification supposedly relies on comparative genomic analyses across tumor samples to determine which mutations recur most frequently, with the most prevalent alteration classified as the driver mutation [1,2,3,4,5].

However, if an initial metabolic defect arises from multiple possible mutations, each inducing a similar metabolic disruption, the assumption that malignant transformation occurs as a linear progression from genetic alteration may be flawed [4]. Here, we hypothesize that this scenario underlies the observed discrepancies between genetic and metabolic models of carcinogenesis.

## 2. An Alternate Hypothesis for Carcinogenesis

**Figure 1:**
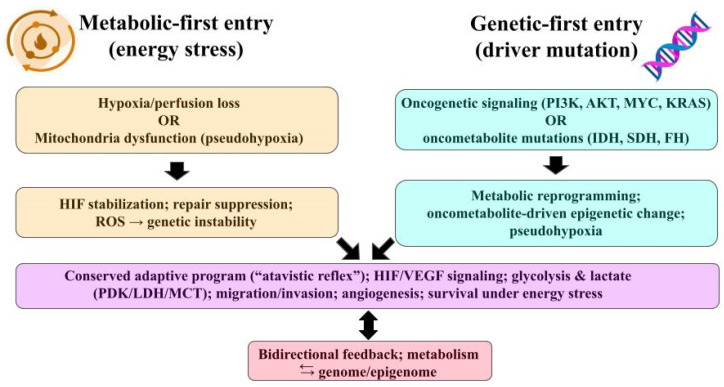
Two entry points into the atavistic reflex. **Left**—Metabolic-first: hypoxia/perfusion loss or mitochondrial dysfunction (pseudohypoxia) → HIF stabilization, DNA-repair suppression, ROS → genomic instability. **Right**—Genetic-first: oncogenic signaling (PI3K/AKT, MYC, KRAS) or oncometabolic mutations (IDH/SDH/FH) → metabolic reprogramming and epigenetic change. Both paths converge on a conserved adaptive program (HIF/VEGF; glycolysis & lactate axes—PDK/LDH/MCT; migration/invasion; angiogenesis; survival under energy stress) with bidirectional feedback between metabolism and the genome/epigenome. Abbreviations: HIF, hypoxia-inducible factor; VEGF, vascular endothelial growth factor; PDK, pyruvate dehydrogenase kinase; LDH, lactate dehydrogenase; MCT, monocarboxylate transporter; ROS, reactive oxygen species; PI3K, phosphatidylinositol 3-kinase; AKT, protein kinase B, MYC, MYC proto-oncogene; KRAS, Kirsten rat sarcoma viral oncogene; IDH, isocitrate dehydrogenase; SDH, succinate dehydrogenase; FH, fumarate hydratase.

Carcinogenesis can commence through two primary routes (Figure 1). A metabolic-first approach, where cellular energy stress, such as hypoxia or mitochondrial dysfunction, leads to genomic instability [18], or a genetic-first mechanism, characterized by oncogenic or “oncometabolic” mutations that instigate metabolic reprogramming [19]. Both of these pathways swiftly establish a bidirectional feedback loop between cellular metabolism and the genome or epigenome [20].

In metabolic-first trajectory (Figure 1), a transient or chronic hypoxia/ischemia and mitochondrial dysfunction can stabilize hypoxia-inducible factor (HIF) signaling, impair DNA repair programs, and increase mutational burden through reactive oxygen species (ROS) and repair suppression. This can provide the substrate for subsequent selection of oncogenic clones. This sequence is supported by reviews showing hypoxia-driven genomic instability and mitochondria-to-nucleus ROS signaling that accelerates acquisition of additional mutations [21,22].

In genetic-first trajectory (Figure 1), canonical oncogenic events (e.g., PI3K/AKT, MYC, KRAS) directly reprogram metabolism to support biomass synthesis and redox balance. Conversely, oncometabolic mutations (isocitrate dehydrogenase (IDH1/2), succinate dehydrogenase (SDH), fumarate hydratase (FH)) create metabolites (2-hydroxyglutarate (2-HG), succinate, fumarate) that inhibit α-ketoglutarate (KG)–dependent dioxygenases, remodel the epigenome, and can stabilize HIF (i.e., pseudohypoxia), placing metabolism upstream of broad transcriptional change even though the initiating hit is genetic [23,24].

After either entry point, selection favors clones, whose metabolic program fit the prevailing nutrient and oxygen constraints. Hypoxia and oncometabolites further establish epigenetic change and genomic instability, producing the well-known metabolism–genome feedback loop [25,26].

**Figure 2:**
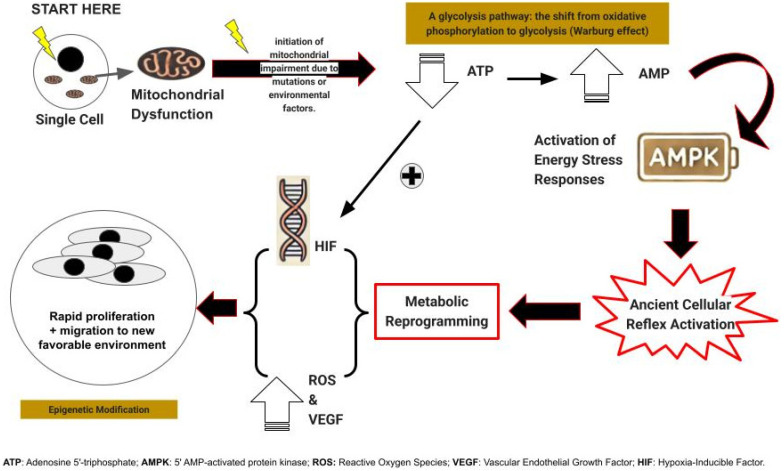
Summarizes the metabolic first trajectory of carcinogenesis.

It may, at first glance, seem paradoxical that cells facing an abrupt loss of available energy would respond by proliferating and migrating, both energy-intensive processes, yet this is precisely what we propose (Figure 2).

First, as a response to sudden energy depletion, unicellular precursors to modern multicellular organisms react by rapid cell proliferation and migration, strategies designed to generate progeny better equipped to thrive in the altered environment or to seek out more hospitable surroundings [27].

In multicellular organisms, this ancient reflex, while potentially detrimental, was nonetheless conserved if it served a critical function, most notably wound healing [28]. However, its expression came under stringent regulatory control and underwent further evolution, including the induction of vascular endothelial growth factor (VEGF) [28]. This response is generated or augmented by adjacent cells undergoing this transformation, resulting in the copious production of lactate or its metabolites, pyruvate, and purines [29].

In a subset of tumors, early initiating events affect TCA enzymes or mitochondrial pathways, notably IDH1/2 (2-HG), SDH, and FH, whose oncometabolites inhibit α-KG–dependent dioxygenases, stabilize HIF, and remodel the epigenome. In many other cancers, metabolic reprogramming follows oncogenic or tumor-suppressor alterations rather than preceding them [30].

These oncometabolites (2-HG, succinate, fumarate) play a crucial role in promoting cancer cell survival and plasticity, especially under conditions of energy stress [31]. They accumulate due to mutations in TCA enzymes or mitochondrial pathways [31], which leads to dysregulated signaling pathways that create an environment conducive to cancer development [31].

Moreover, mitochondrial DNA (mtDNA) is a hotbed for mutations in cancer, largely due to its susceptibility to oxidative damage and limited repair mechanisms. These vulnerabilities frequently lead to disruptions in the electron transport chain (ETC) pathway.

The ensuing selective pressures then favor and establish those mtDNA variants that best support the cancer cell’s immediate environment and survival. These factors explain enrichment of TCA/mitochondrial lesions in specific tumor types (e.g., IDH-mutant glioma/acute myeloid leukemia, SDHx in pheochromocytoma/paraganglioma, and FH-deficient renal cell carcinoma) without implying a universal rule [32,33].

The failure of one of TCA enzymes often precipitates the failure of all. As a consequence of this enzymatic collapse, oxidative phosphorylation and metabolism falter, leading to an abrupt decline in cellular energy production. The affected cell can then derive a mere two energy units [adenosine triphosphate (ATP) molecules] per glucose molecule, a stark contrast to the thirty-six or thirty-eight ATPs typically generated. This metabolic shift is accompanied by a marked increase in low-energy adenosine monophosphate (AMP) [34]. The “abrupt loss of energy” response is therefore reactivated.

This process is accelerated by the increased AMP levels activating AMP-activated protein kinase (AMPK) [34], triggering VEGF production and cellular proliferation through a mechanism that may be somewhat “sloppy,” leading to the emergence of numerous mutant cells [28]. These cells simultaneously undergo migration, a phenomenon recognized in tumor cells as invasion [35].

Sooner or later, these cells manifest nuclear atypia, a defining characteristic of malignant cells and their precursors. Subsequently, these cancer cells exhibit mutations in cell proliferation and control genes, later identified as “driver mutations” [36].

However, mutations in TCA cycle genes or mitochondrial ETC genes are less readily recognized as driver mutations, primarily because any of a multitude of these genes may be mutated. These mutations lead to the accumulation of oncometabolites resulting in epigenetic alterations and a pseudohypoxic state that promotes tumorigenesis [37].

Beyond glucose metabolism, cancer cells often exhibit alterations in amino acid metabolism, which are crucial for supporting rapid proliferation and survival. Amino acids such as glutamine, arginine, tryptophan, asparagine, and aspartate play significant roles in tumor growth and modulating the tumor microenvironment [38]. Interactions between amino acid metabolism and signaling molecules like VEGF and HIF-1α further influence tumor progression and angiogenesis.

Additionally, tumor-associated macrophages (TAMs) contribute to the immunosuppressive microenvironment through metabolic reprogramming, including altered amino acid metabolism, which supports tumor growth and metastasis.

Therefore, the collapse of the TCA cycle not only forces cells to rely on glycolysis but also drives compensatory metabolic reprograming, notably involving amino acid pathways such as glutaminolysis and asparagine metabolism. These pathways can replenish TCA intermediates (anaplerosis) and sustain redox balance, allowing cells to partially mitigate energy deficits and maintain biosynthesis, thus linking TCA failure directly to the upregulation of amino acid metabolism.

## 3. Manifestations of the Atavistic Reflex in Modern Multicellular Organisms

While we cannot fully reconstruct the environmental conditions of our unicellular ancestors, we can identify remnants of their adaptive responses that persist in modern multicellular organisms [39]. We propose that certain physiological manifestations reflect an evolutionarily conserved “abrupt loss of energy” reflex. Some of these responses may confer benefits, while others contribute to pathological processes.

One such example is the rapid mitochondrial dysfunction leading to an abrupt decline in ATP production. This shift forces the cell to transition from oxidative phosphorylation to glycolysis, resulting in lactate accumulation. Lactate is subsequently converted into pyruvate and alanine, a non-essential amino acid. Fluctuations in these metabolites may trigger or amplify the “abrupt loss of energy” reflex through distinct regulatory mechanisms, potentially influencing carcinogenic processes.

Important players in this mechanism are HIFs, which are transcription factors that regulate cellular responses to decreased oxygen availability. It has been recognized for many years that hypoxia is a well-documented feature of various neoplasms, traditionally attributed to inadequate vascularization [40]. However, HIFs also respond to pseudohypoxia; oxygen-independent activation triggered by factors such as thiamine deficiency or mutations affecting TCA cycle enzymes or mitochondrial oxygen transport genes [41]. Both hypoxia and pseudohypoxia rapidly impair mitochondrial ATP production, potentially initiating this metabolic response [42].

In addition, AMP-activated protein kinase (AMPK) functions as a cellular energy sensor, and it is activated under conditions of low ATP levels [31]. In specific contexts, AMPK activation can facilitate cancer cell survival and proliferation under metabolic stress, highlighting its dual role in carcinogenesis [43]. Indeed, AMPK activation promotes ATP production by increasing catabolism while decreasing anabolism [43]. This response serves to balance energy production and consumption.

Moreover, glycolysis produces abundant lactate and its metabolites, pyruvate and alanine [13]. These metabolites engage in a complex network of cellular signaling, including reinforcement of the abrupt loss of energy reflex, amplifying the ongoing metabolic response [44]. As loss of muscle pyruvate dehydrogenase (PDH) demonstrates, this rapid increase in glycolysis and subsequent accumulation of pyruvate and alanine contributes to severe lactic acidosis [44]. This, then, becomes a self-perpetuating cycle, reminiscent of a runaway train, underscoring the significance of these metabolites in modulating cellular responses to energy stress.

## 4. Tissue Proliferation as Atavistic Clues to Carcinogenesis

The concept of carcinogenesis, as the misfiring of an ancient survival mechanism, could offer a unifying explanation for the metabolic and genetic chaos that defines cancer [45]. Furthermore, this perspective can provide a framework to understand a spectrum of pathological conditions and suggest clues to carcinogenesis.

The pathologic proliferation, that occurs in diverse diseases, appears to be driven by the atavistic loss-of-energy reflex, which was originally an adaptive response to hypoxia and metabolic stress [45]. This conserved mechanism enables cells to survive energy deprivation by initiating proliferation and migration; however, in the complex environment of modern multicellular organisms, this reflex can manifest in maladaptive ways, contributing to disease pathogenesis rather than promoting survival [45].

**Figure 3:**
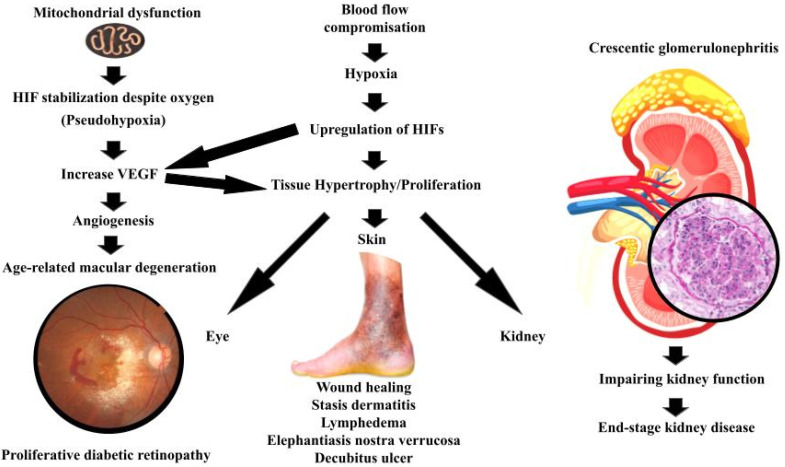
Summarizes the manifestation of the atavistic reflex in different organs.

It may be that certain physiological manifestations reflect an evolutionarily conserved “abrupt loss of energy” reflex, with the balance between benefit and harm often determined by the specific tissue context and the degree of regulatory control [45]. Figure 3 and Table 1 summarize this concept by exploring different diseases grouped based on the initiating mechanism.

### 4.1. Hypoxia-Driven Proliferation (True Ischemia)

The commonly understood mechanism is that reduced oxygen tension from impaired perfusion and pressure will lead to increase HIF-1α and VEGF, which leads to proliferation and migration. Numerous studies have noted that oxygen tension in healing wounds is significantly reduced [45]. In this context, the proliferation and migration of fibroblasts to the wound site, a key step in tissue repair, may be driven by the activation of this atavistic reflex [46].

Fibroblasts are essential for the formation of granulation tissue [46]. They migrate into the wound area and deposit matrix de novo, with their migration and proliferation rate-limiting steps to repair wounds [47]. It may be that this ancient survival response, vital for the survival of multicellular organisms, is necessarily preserved, even if it carries the risk of dysregulation [48].

In lymphedema and elephantiasis nostra verrucosa, areas with compromised lymphatic drainage and poor oxygen perfusion, and therefore reduced oxygenation, exhibit tissue hypertrophy [45]. As a result of this oxygen deficit, the skin undergoes massive hyperplasia, paradoxically increasing the tissue’s oxygen demand in an area already struggling with hypoxia [49]. The chronic, progressive accumulation of protein-rich fluid within the interstitium and the fibro-adipose tissue exceeds the capacity of the lymphatic system to transport the fluid [50]. This creates a vicious cycle, wherein the initial insult of reduced blood flow triggers a proliferative response that ultimately exacerbates the underlying oxygen deficiency [49].

Stasis dermatitis presents a seemingly contradictory picture. While blood flow in affected areas is often increased, the permeability of blood vessels is markedly reduced, leading to decreased oxygen tension in the tissues [51]. This reduced oxygenation triggers hyperplasia of the affected skin, further compromising oxygen delivery, and, in some cases, leading to the more severe manifestation of elephantiasis nostra verrucosa [45]. If left untreated, the condition can lead to more serious conditions including venous ulcerations [51]. As such, specific oxygen dosing as a function of tissue hypoxia is key to a successful outcome [52].

In decubitus ulcers, where sustained pressure deprives the skin of blood flow, tissue dies over time. Examination of these lesions reveals that the surrounding skin often exhibits marked hypertrophy, driven by increased cell proliferation [53]. This hypertrophic response, while perhaps initially having the effect of protecting the underlying tissue, increases the oxygen demand in an area already suffering from severe ischemia, ultimately contributing to the formation of deep, necrotic ulcers [53]. As a result, current research has suggested that decreasing pressure and keeping skin clean, while avoiding scrubbing can reduce these ulcers [53].

Lastly, proliferative diabetic retinopathy (PDR), a complication of advanced diabetes mellitus (DM), provides another compelling example [54]. In DM-associated PDR, restricted blood flow through small vessels supplying oxygen to the retina leads to hypoxia [55]. This hypoxia, in turn, drives the proliferation of non-light reactive cells, ultimately leading to blindness as these cells obscure the photoreceptors [56]. It may be that hypoxia-driven pathways are involved, paralleling mechanisms observed in cancer and age-related macular degeneration (AMD), reflecting the conserved nature of the cellular response to energy deprivation [45].

### 4.2. Pseudohypoxia and Mitochondrial Dysfunction (Metabolic)

The main mechanism is that TCA and ETC defects or mitochondrial injury leads to increase oncometabolites, PHD inhibition, HIF stabilization despite oxygen (pseudohypoxia), and increase VEGF, which leads to proliferation and angiogenesis.

One example that illustrates this process is age-related macular degeneration (AMD). Although AMD is not a neoplastic condition, it exemplifies how hypoxia-induced VEGF expression, often driven by underlying mitochondrial dysfunction and energy deprivation, can result in pathological angiogenesis. This parallel reinforces our central hypothesis, that the atavistic ‘loss-of-energy’ reflex, triggered by metabolic disruption and hypoxia, may drive cellular behaviors such as proliferation and angiogenesis in various pathologies, including but not limited to cancer.

As a leading cause of vision loss in the elderly, AMD particularly highlights this complex interplay [57]. The neovascular (wet) form of AMD involves abnormal blood vessel growth in the retina, driven, in part, by hypoxia-induced expression of VEGF [58]. VEGF promotes angiogenesis in the initial stage of choroidal neovascularization that further leads to increased vascular permeability [58]. Evidence suggests that mitochondrial dysfunction, specifically mitochondrial DNA damage and dysfunction in retinal pigment epithelial cells, plays a key role in AMD [57].

This mitochondrial impairment leads to energy deficits and oxidative stress, further contributing to disease progression [57]. Hypoxia in retinal cells leads to the stabilization of HIFs, which, in turn, upregulate VEGF expression [56]. VEGF promotes angiogenesis, increasing oxygen supply but also leading to pathological changes, a balance between benefit and harm, which is central to our hypothesis [56]. Indeed, anti-VEGF therapies have proven effective in treating wet AMD, underscoring the role of hypoxia-induced metabolic changes in driving the disease [56].

Additionally, in certain severe forms of glomerulonephritis, most notably crescentic glomerulonephritis, a striking proliferation of parietal epithelial cells occurs along Bowman’s capsule [54]. This proliferation, which occupies Bowman’s space, gives rise to cellular crescents that compress the glomerular tuft, ultimately impairing kidney function [59], and often leads to end-stage kidney disease [60].

In crescentic glomerulonephritis, inflammation leads to occlusion of capillaries and disruption of normal blood flow. This results in a state of hypoxia and energy deprivation in glomerular cells, particularly the parietal epithelial cells lining Bowman’s capsule. The response to this deprivation includes the upregulation of HIFs. It may well be that the hypoxic environment and energy scarcity within the glomerulus activate an ancient, atavistic cellular reaction, prompting proliferation. In this scenario, the parietal epithelial cells of Bowman’s capsule respond to energy deprivation by proliferating and migrating into the urinary space, forming crescents [59].

In effect, this process mirrors the proposed mechanism wherein cells, facing a sudden energetic crisis, revert to a more primitive survival strategy, prioritizing proliferation and migration, even at the expense of normal function. Support for this perspective comes from a variety of sources. The increased expression of HIF-1α and VEGF in glomeruli affected by crescentic glomerulonephritis suggests the involvement of hypoxia-driven pathways in these pathological changes [60].

Moreover, studies indicating mitochondrial damage in glomerular cells during severe glomerulonephritis point to decreased ATP production and increased ROS generation, thereby underscoring the role of metabolic dysfunction in initiating cellular responses. Perhaps most compelling is the evidence that experimental treatments designed to reduce hypoxia or inhibit HIFs and VEGF have shown promise in ameliorating glomerular injury and proliferation in animal models [61,62]. Further investigation of such interventions may prove invaluable.

### 4.3. Stress-Triggered Reflex Reactivation (UV/AK)

The principal mechanism is that ultraviolet (UV)-induced cellular stress and DNA damage create local energy stress, which activate migration and proliferation programs reminiscent of early neoplastic invasion. Actinic keratoses (AKs) are characterized by keratinocytes mutated by chronic sunlight damage [63]. These lesions carry the potential to evolve into squamous cell carcinoma, a distinctly malignant entity [63].

Stressed in modern dermatopathology textbooks, AK keratinocytes, though not themselves malignant, exhibit a tendency for affected epidermal cells to migrate into the underlying dermis [64,65].

It may be that this migratory behavior, reminiscent of the invasive properties of cancer cells, represents another manifestation of the atavistic loss-of-energy reflex, reactivated by the cellular stress induced by UV radiation [63].

## 5. Metabolic Reprogramming in Cancer

It has become increasingly clear that cancer cells, in their relentless pursuit of unchecked proliferation, exhibit a remarkable plasticity in their metabolic strategies. This metabolic dysregulation is hallmarked by a markedly increased glucose uptake, coupled with what some might term a rather profligate production of lactate, even when oxygen is readily available [66]. This preference for anerobic glycolysis, a phenomenon often referred to as the Warburg effect, is not merely an inefficient means of energy production; rather, it is a carefully orchestrated metabolic reprogramming that serves the biosynthetic and energy requirements of rapidly dividing cancer cells [13].

Emerging evidence suggests that mitochondrial dysfunction may play a more pivotal role in cancer development than previously appreciated, potentially acting as an instigator of genomic instability. Recent studies have illuminated that mitochondrial dysfunction can precede and, perhaps more alarmingly, actively promote genetic mutations [67]. Defects in mitochondrial respiration can unleash a torrent of ROS, those molecular wrecking balls that inflict damage upon DNA and incite genomic instability [67].

In cancer, HIFs are often found to be upregulated, not solely as a response to hypoxia within tumors, but also as a consequence of mitochondrial dysfunction [68]. This aberrant activation of HIFs triggers an increased expression of VEGF and other factors that promote angiogenesis, glucose metabolism, and cell survival, all of which contribute to the insidious growth and metastatic spread of tumors [68]. These upregulated factors create an environment ripe for tumor progression.

Perhaps one of the most intriguing aspects of cancer metabolism is the phenomenon of pseudohypoxia, a state in which cells behave as if they are starved of oxygen, even when oxygen is readily available [69].

Mutations in key enzymes of the TCA cycle, such as SDH and FH, can lead to the accumulation of succinate and fumarate, respectively [69]. These oncometabolites, as they are sometimes called, inhibit PHD, the very enzymes that regulate HIF degradation, leading to HIF stabilization even under normoxic conditions [69].

The resulting aberrant activation of HIFs then promotes oncogenic pathways that fuel the relentless growth and spread of cancer [69]. Therefore, several genes are upregulated, including glycolysis (e.g., LDHA, GLUT1, PDK1), angiogenesis (VEGF), and survival pathways [70,71,72]. This contributes to therapy resistance and tumor progression.

Some therapeutic approaches discussed in the literature include direct HIF inhibition (e.g., PX-478 inhibits HIF-1α deubiquitination. In addition, PT2385 and Belzutifan selectively antagonize HIF-2α, disrupting dimerization and transcriptional activity) [73].

Another strategy includes targeting metabolic enzymes, such as dichloroacetate (DCA), which inhibits PDK1, reversing glycolysis [74], IDH mutant inhibitors (e.g., Enasidenib, Ivosidenib), which decrease 2-HG levels and restore PHD function [75], or LDH and monocarboxylate transporter (MCT) inhibitors, which impair lactate flux and reduce immune suppression [76].

Although primarily developed for hypoxic tumors, these strategies also mitigate pseudohypoxia by improving oxygen sensing and metabolism [72]. Given the metabolic plasticity of cancer cells, monotherapy may lead to compensatory adaptations. Therefore, combination therapies targeting HIFs, metabolic pathways, and the tumor microenvironment may enhance efficacy [77]. Additionally, the integration of these strategies with immunotherapy (e.g., immune checkpoint inhibitors) may overcome immune evasion in pseudohypoxic tumors [78].

Mutations in mitochondrial DNA (mtDNA) are commonplace in the chaotic landscape of cancer [79]. These mutations can wreak havoc on oxidative phosphorylation complexes, leading to a dysfunction of the respiratory chain [79]. Somatic mtDNA mutations can affect the functions of tRNA [tRNAVal (T1659C), tRNAAla (G5650A)], NADH dehydrogenase [ND1 (G3842A), ND4 (11032delA, A11708G), ND5 (12418insA)], and Cytochrome C oxidase [COI (T6787C), COII (G7976A), COIII (A9263G, G9267A)] [79].

In the face of mitochondrial dysfunction, cancer cells are compelled to rely on glycolysis for their survival [13]. This reliance on glycolysis, known as the Warburg effect, provides the metabolic intermediates necessary for biosynthesis, which are the essential building blocks that fuel the rapid cell proliferation and ensure survival under the harsh conditions that prevail within the tumor microenvironment [13].

Mitochondrial dysfunction often begets a surge in the production of ROS [67]. These ROS can inflict damage upon DNA, proteins, and lipids, thereby contributing to genomic instability [67].

## 6. Epigenetic Consequences of Metabolic Dysfunction

The metabolic state is connected into chromatin by enzymes that use metabolites as substrates or cofactors. Fluctuations in oxygen or metabolite pools therefore translate directly into lasting alterations in gene regulation.

Ten-eleven translocation (TET) DNA demethylases and Jumonji C (JmjC) histone demethylases require α-KG, Fe(II), and O_2_. Accumulation of oncometabolites such as 2-HG, succinate, or fumarate competitively inhibits these enzymes, producing widespread DNA and histone hypermethylation and blocking differentiation programs. Hypoxia independently limits dioxygenase activity by depriving oxygen. Together, these mechanisms create a hypermethylated epigenetic state that stabilizes oncogenic transcriptional circuits [80,81].

DNA and histone methyltransferases consume S-adenosyl-methionine (SAM), while S-adenosyl-homocysteine (SAH) inhibits them; the cellular SAM:SAH ratio (“methylation index”) therefore gates methylation potential. Nutrient flux through the folate/methionine cycle (serine/glycine availability, methionine supply, B-vitamin cofactors) rapidly alters histone methylation (e.g., H3K4me3) and gene expression, linking diet and tumor nutrient stress to chromatin state [82].

Histone acetylation tracks local nuclear acetyl-CoA. Glucose-citrate–derived acetyl-CoA via ATP-citrate lyase (ACLY) supports global acetylation; under stress, nuclear pyruvate dehydrogenase complex (PDC) and nuclear acetyl-CoA synthetase 2 (ACSS2) can generate promoter-proximal acetyl-CoA to maintain transcription of stress-adaptation programs (e.g., lysosome/autophagy genes). Thus, metabolic reprograming that shifts acetyl-CoA sourcing reshapes chromatin accessibility [83,84].

Sirtuins are NAD^+^-dependent deacetylases/deacylases that compact chromatin and enforce genome stability; PARP-mediated DNA repair also consumes NAD^+^. Shifts in NAD^+^ availability-through redox changes, DNA damage, or consumption by PARPs/CD38-alter sirtuin activity and thereby histone acetylation and transcriptional programs [85].

Beyond acetylation, diverse acyl-CoA species and metabolites imprint chromatin. Lactate directly installs histone lactylation, an activation-linked mark induced by hypoxia/high glycolysis and observed in tumor and immune cells. β-hydroxybutyrate drives histone β-hydroxybutyrylation under ketogenic/oxidative conditions. These marks provide additional routes by which metabolic states are read at chromatin [86,87].

The hexosamine biosynthetic pathway integrates glucose, amino acids, and lipids to produce UDP-GlcNAc for O-GlcNAc transferase (OGT). O-GlcNAcylation modifies histones and chromatin regulators (including crosstalk with TET and Polycomb complexes), altering chromatin compaction and transcription in response to nutrient status; a mechanism heightened in cancer metabolism [88].

Hypoxia and oncometabolite accumulation tilt the balance toward DNA/histone hypermethylation; nutrient and redox constraints reshape acetylation and deacetylation; and newly recognized acyl/sugar modifications encode additional layers of metabolic memory on chromatin. Collectively, metabolic dysfunction reprograms the epigenome to sustain malignant phenotypes (dedifferentiation, therapy resistance, and immune modulation) [25].

## 7. Additional Supporting Evidence: Stem Cells, Autophagy, and AMPK

Ito and Suda [89] have shown that hypoxic conditions and metabolic stress can maintain the quiescence of hematopoietic stem cells, while shifts in metabolism can propel them toward proliferation and differentiation. The dysregulation of these finely tuned processes can lead to the uncontrolled proliferation of cells, as seen in leukemogenesis.

Autophagy has emerged as a key player in cancer metabolism [90]. Under conditions of metabolic stress, autophagy can provide a lifeline to cells, supplying them with essential nutrients by recycling cellular components [90]. However, if this process is dysregulated, it can also contribute to the initiation and progression of cancer [90]. An important potential addition to the autophagic process would be its ability to provide a link to the tumor microenvironment [13].

Adenosine monophosphate-activated protein kinase (AMPK) is activated when ATP levels plummet, triggering a cascade of events aimed at restoring energy balance [91]. Activation of AMPK can inhibit cell proliferation by modulating metabolic pathways, essentially putting the brakes on uncontrolled growth [91]. However, it is important to consider that AMPK activation, in certain contexts, can also promote cancer cell survival under metabolic stress, suggesting that its role in carcinogenesis is far more complex than a simple good-versus-evil dichotomy [91].

Across more than 1000 tumors with whole-genome data, elevated hypoxia scores are linked to higher mutational load and copy-number aberration, with enrichment of TP53/MYC/PTEN driver alterations and worse clinical behavior is consistent with hypoxia shaping tumor evolution [92].

Individual-patient meta-analysis and prospective series show ^18^F-fluoromisonidazole)/^18^F-fluoroazomycin arabinoside (FMISO/FAZA) PET–measured hypoxia is independently associated with poorer locoregional control and survival in head-and-neck cancer [93,94]. In addition, persistent hypoxia during chemoradiation portends adverse outcomes [93,94].

Meta-analyses report that high Glucose-Transporter-1 (GLUT1) expression (a hypoxia/glycolysis marker) correlates with inferior overall survival across cancers; Carbonic Anhydrase IX (CAIX), an established hypoxia-induced pH regulator, is likewise associated with adverse prognosis in many solid tumors (with some tumor-specific exceptions) [95].

In IDH-mutant glioma, mutant IDH is sufficient to establish the G-CIMP hypermethylator phenotype in patient-derived contexts. Human SDH-mutant paraganglioma/pheochromocytoma and FH-deficient renal cancers show SDH/FH-linked hypermethylation and pseudohypoxia signatures, directly tying mitochondrial TCA lesions to epigenetic remodeling in vivo [30,96].

Patient datasets and meta-analyses link higher hypoxia-inducible metabolic enzymes/transporters (LDHA, PDK1, and MCT4/SLC16A3) expression to worse outcomes in multiple cancers, consistent with lactate-supported flux reinforcing the program [97,98].

In retinal neovascular disease -the same hypoxia-driven arm emphasized in our model-randomized trials show anti-VEGF improves vision in neovascular AMD and is non-inferior to panretinal photocoagulation for proliferative diabetic retinopathy [99,100].

Taken together, omics data reinforce that oxygen and energy stress activate a common metabolic–epigenetic program, providing a mechanistic basis for the observed clinical associations with prognosis and therapeutic response [92].

## 8. Animal Models and Human Trials Targeting Metabolic Pathways

Animal models have provided compelling evidence linking metabolic dysfunction to cancer [101]. For instance, it has been demonstrated that mice with mutations in the mitochondrial polymerase gamma (POLG) gene, which is responsible for replicating mtDNA, accumulate mtDNA mutations and exhibit increased tumor formation [67]. Although the majority of evidence supports a role of mtDNA mutations in tumorigenesis and malignant progression, much investigation is required before we can establish direct causality of mtDNA mutations in carcinogenesis [101].

*PHD2^+/−^* mice model showed that endothelial oxygen sensing leads to vascular normalization. PHD2 haplodeficiency in endothelium normalizes tumor vessels, improves oxygenation, and reduces metastasis, showing that relieving hypoxia suppresses malignant spread [102]. IDH1-R132H mouse glioma models produce 2-HG and G-CIMP–like epigenetic remodeling, supporting a genetic-first driver of metabolic/epigenetic route [103].

Fh1-deficient kidney models show profound metabolic connectivity (KEAP1/Nrf2 activation; cysts/tumorigenesis), illustrating pseudohypoxic/metabolic entry, even where some phenotypes are HIF-independent, which underscores pathway diversity in energy-stress responses [30].

Finally, genetic or pharmacologic LDH/MCT fluctuations in mice reduces tumor growth or alters immune milieu in multiple systems, consistent with our metabolite-amplified energy stress axis (with context-dependent exceptions) [104].

These findings underscore the notion that tinkering with the delicate metabolic balance within cells can have profound consequences for their propensity to develop into tumors. The insights gleaned from preclinical studies have spurred a wave of clinical trials exploring therapies that target cancer metabolism directly [105].

Inhibitors of IDH mutations have shown promise in treating certain leukemias and gliomas, offering a glimpse of the therapeutic potential of targeting cancer’s metabolic underbelly [105].

Metformin, a drug known for its ability to activate AMPK, is also under investigation for its anticancer properties, owing to its multifaceted effects on cellular metabolism [93].

HIF-2α inhibition (belzutifan) directly targets pseudohypoxia. It was FDA-approved in 2021 for VHL-associated tumors, and in 2023 for advanced renal cell carcinoma after prior lines. Outcomes include improved progression free survival (PFS) vs. everolimus in a pivotal study with a favorable safety profile. It supports that blocking the hypoxia transcriptional arm has clinical benefit in pseudohypoxic RCC [106].

In addition, FDA approved in 2024 mutant-IDH inhibition (vorasidenib). INDIGO phase 3 in IDH-mutant grade-2 glioma showed PFS [27.7 vs. 11.1 months] (HR 0.39), and delayed time to next intervention compared to placebo group. This validates that oncometabolite production and downstream epigenetic/metabolic reprogramming are actionable in humans [107].

Lactate transport blockade (AZD3965 or monocarboxylate transporter 1 (MCT1) inhibitor) in phase I first-in-human showed target engagement and preliminary activity. The expansion cohorts in DLBCL/Burkitt have been selected by MCT1-high/MCT4-low biology. It represents a clinical proof-of-mechanism for the lactate axis our model highlights [108].

In terms of hypoxia-activated prodrugs (evofosfamide/TH-302; tirapazamine), despite strong preclinical hypoxia-selective targeting, phase III trials failed to improve survival in common settings. This likely reflects tumor hypoxia heterogeneity or patient selection. These results propose that biomarker-guided hypoxia targeting (imaging/transcriptomics) could be essential [109,110,111].

Finaly, mitochondrial flux modulators (PDK or TCA targeting), especially dichloroacetate (PDK inhibitor) has shown feasibility and biomarker-informed dosing (early-phase; mixed signals). However, devimistat (CPI-613) has failed in phase III pancreas cancer, underscoring the need for mechanism-matched selection [112].

Therefore, the success of these interventions is often dependent on the patient population and specific type of cancer [107].

## 9. Implications for Cancer and Beyond

### 9.1. Implications for Cancer

Framing carcinogenesis as a process initiated by metabolic dysfunction unlocks new avenues for both the prevention and treatment of this insidious disease. As such, this reframing presents several potential therapeutic targets. Preclinical and clinical data suggest that targeting mitochondrial dysfunction, glycolytic flux, or redox imbalance can disrupt cancer’s metabolic foundation [113]. Epigenetic remodeling induced by oncometabolites further highlights a therapeutic window, as seen with mutant-IDH inhibition [114]. Strategies that limit ROS-driven DNA instability may complement conventional therapies by slowing mutation accumulation [115]. Integrating metabolic inhibitors with traditional chemotherapy or targeted therapies could enhance treatment efficacy by simultaneously attacking cancer cells from multiple angles.

However, although the atavistic reflex may represent a conserved response to metabolic stress, its manifestation likely varies with the tissue type, genetic background, and degree of mitochondrial dysfunction. This may explain why only certain metabolic drugs (e.g., IDH inhibitors, metformin) show efficacy, highlighting the need for individualized metabolic profiling before therapy selection.

The atavistic reflex hypothesis may also inform biomarker development. Metabolic markers such as HIF-1α, VEGF, lactate, and AMPK activation may serve not just as hallmarks of energy stress but as predictive biomarkers guiding stratified therapy.

### 9.2. Implications Beyond Cancer

The metabolic responses proposed herein may also be relevant to understanding, and therapeutic intervention, in other human or animal diseases. For instance, phocomelia, which is a rare congenital anomaly characterized by the absence of intermediate segments of the extremity [116], may respond to site-directed pyruvate injections, perhaps even delivered in utero [117]. Similarly, one can envision a future in which we are able to regenerate organs damaged by disease or injury, thereby obviating the need for the often-difficult and traumatic process of organ transplantation [116].

## 10. Obesity, Sugary Food/Drink, and Cancer

Recent evidence has increasingly suggested that elevated serum glucose levels, as seen in obesity and the overconsumption of sugary foods or beverages, may function as a key metabolic component predisposing individual to the development of cancer [118]. Indeed, this association has been observed in at least twelve human internal cancers, comprising a staggering 40 percent of all internal human cancers, results that have been further corroborated in animal models [119].

While the precise etiopathogenesis remains to be fully elucidated, we can envision several potential mechanisms at play. Perhaps the most straightforward explanation is that elevated glucose levels provide a rich and readily available fuel source for those glucose-hungry cancer cells, accelerating their proliferation and fueling their relentless expansion [119].

High dietary glycemic load raises insulin/insulin growth factor (IGF-1), activating PI3K–AKT–mTOR signaling pathway programs, one of the central growth and survival pathways in cells. This activation increases glucose uptake and anabolic flux, which are metabolic changes that align with our loss-of-energy model’s glycolysis-amplified arm. Epidemiologically, glycemic index/glycemic load (GI/GL) show weak-to-moderate associations with risk in select cancers (e.g., endometrium; mixed across sites). Mechanistically, glucose metabolism supplies citrate, which is converted by ATP-citrate lyase into acetyl-CoA that fuels histone acetylation, thereby linking nutrient availability to chromatin accessibility and gene expression [120,121].

It is also possible that the loss of a TCA cycle enzyme or a gene controlling mitochondrial ETC could lead to a more abrupt decrease in energy and trigger that aforementioned atavistic reflex [119].

Other important factor is high-fat diet (HFD), which can augment intestinal stem-cell number and function via PPAR-δ signaling, increasing tumorigenic potential in vivo [122]. Independently, fatty-acid uptake via CD36 supports the function of metastasis-initiating cells. Experimental evidence shows that palmitate exposure or HFD enhances metastasis in mouse models, whereas CD36 inhibition suppresses dissemination [122]. These data situate dietary lipids as fuel and signal, supporting energy homeostasis, redox balance, and metastatic programs within our framework [122].

Therefore, high sugar availability channels metabolism through glycolysis and acetyl-CoA production, promoting chromatin acetylation and anabolic growth, whereas lipid metabolism supplies acetyl-CoA via fatty-acid oxidation and activates signaling pathways that enhance stemness and metastatic potential. Both inputs can reinforce the conserved energy-stress reflex (HIF/VEGF and metabolite amplification), albeit with tumor-type variability and modest effect sizes at the population level [84].

## 11. Addressing Counterarguments

It is certainly true that driver mutations in cell proliferation genes are frequently observed in cancers, a point often raised by those skeptical of the primacy of metabolic dysfunction in tumorigenesis. However, these mutations may well be secondary events, downstream consequences of the initial metabolic dysregulation, rather than the primary instigators of cancer [123]. The sheer diversity of mutations in metabolic genes, and the relatively small role of each mutation, makes them less apparent in genomic studies that focus on recurrent mutations [123].

Skeptics may highlight the well-documented metabolic flexibility of cancer cells and their ability to reconfigure energy production in response to the shifting conditions of the tumor microenvironment [124]. However, this very plasticity supports our hypothesis. An initial metabolic dysfunction compels cells to activate alternative pathways, enabling them to secure energy and resources, thereby sustaining survival and proliferation. In this view, metabolic adaptability is itself a downstream consequence of the original metabolic insult. Moreover, gene-centric models of cancer initiation fail to adequately explain the widespread presence of the Warburg effect [114].

## 12. Conclusions

It is increasingly plausible that carcinogenesis often originates from subtle yet significant metabolic alterations that create energy stress and activate an evolutionarily conserved atavistic program driving proliferation and migration.

Clinical and experimental evidence across diverse conditions supports the view that hypoxia can induce cell proliferation. Taken together, these observations suggest that metabolic dysregulation may, in some cases, precede the oncogenic and tumor-suppressor mutations traditionally considered hallmarks of cancer.

This perspective does not diminish the importance of genetics, but it suggests that, in certain cancers, genetic alterations may be the cart rather than the horse. However, further investigation into mitochondrial function, metabolic pathways, and hypoxia responses is urgently needed to clarify their roles in pathological cell growth.

Targeting these processes may yield innovative and potentially more effective approaches to cancer prevention and therapy. If this hypothesis proves correct, then cancer should not be viewed solely as a genetic disease, but rather as a metabolic misstep, which is an evolutionary trade-off that reflects the very processes that enabled human survival and adaptation.

## Figures and Tables

**Table 1:** Summarizes the atavistic reflex across diseases beyond cancer based on the initiating mechanism. Abbreviations: HIF: Hypoxia-Inducible Factor; VEGF: Vascular Endothelial Growth Factor; PDR: Proliferative Diabetic Retinopathy; TCA: Tricarboxylic Acid cycle; ETC: Electron Transport Chain; SDH: Succinate Dehydrogenase; FH: Fumarate Hydratase; IDH: Isocitrate Dehydrogenase; AMD: Age-Related Macular Degeneration; RPE: Retinal Pigment Epithelium; GN: Glomerulonephritis; ROS: Reactive Oxygen Species; mtDNA: Mitochondrial DNA; mt dysfx: Mitochondrial dysfunction; UV: Ultraviolet; AK: Actinic Keratosis; ↓pO_2_: Low oxygen tension. ↓: Decrease; ↑: Increase.

Category	Shared Mechanism	Representative Conditions	Canonical Markers/Pathways	Therapeutic Corollaries
**Hypoxia-driven**	Perfusion failure → HIF-1α/VEGF program → proliferation/migration	Wound healing; Lymphedema/ENV; Stasis dermatitis; Decubitus ulcers; PDR	↓pO_2_, HIF-1α↑, VEGF↑	Pressure/edema control; oxygen dosing; anti-VEGF (PDR)
**Pseudohypoxia & mitochondrial dysfunction**	TCA/ETC defects or mitochondrial damage → oncometabolites → PHD inhibition → HIF stabilization	Cancer with SDH/FH/IDH/ETC defects; AMD (RPE mt dysfunction); Crescentic GN (glomerular hypoxia signaling)	SDH/FH↑; HIF-1α↑; VEGF↑; ROS/mtDNA signals	Anti-VEGF; HIF/IDH-targeted agents; mitochondrial support
**Stress-triggered reflex (UV/AK)**	UV-induced cellular energy stress reactivates reflex	AK	HIF/VEGF context-dependent; stress/DNA-damage signals	AK prevention/field therapy; block downstream reflex

## Data Availability

No new data were created or analyzed in this study. Data sharing is not applicable to this article.
